# Isolation and characterization of the C-class *MADS-box* gene involved in the formation of double flowers in Japanese gentian

**DOI:** 10.1186/s12870-015-0569-3

**Published:** 2015-07-17

**Authors:** Takashi Nakatsuka, Misa Saito, Eri Yamada, Kohei Fujita, Noriko Yamagishi, Nobuyuki Yoshikawa, Masahiro Nishihara

**Affiliations:** Graduate School of Agriculture, Shizuoka University, 836 Ohya Suruga-ku, Shizuoka, 422-8529 Japan; Iwate Biotechnology Research Center, 22-174-4 Narita, Kitakami, Iwate 024-0003 Japan; Faculty of Agriculture, Iwate University, 3-18-8 Ueda, Morioka, Iwate 020-8550 Japan

**Keywords:** AGAMOUS, *Apple latent spherical virus* vector, Double-flowers, Japanese gentian, LTR-type retrotransposon, *MADS-box* genes

## Abstract

**Background:**

Generally, double-flowered varieties are more attractive than single-flowered varieties in ornamental plants. Japanese gentian is one of the most popular floricultural plants in Japan, and it is desirable to breed elite double-flowered cultivars. In this study, we attempted to characterize a doubled-flower mutant of Japanese gentian. To identify the gene that causes the double-flowered phenotype in Japanese gentian, we isolated and characterized *MADS-box* genes.

**Results:**

Fourteen *MADS-box* genes were isolated, and two of them were C-class *MADS-box* genes (*GsAG1* and *GsAG2*). Both *GsAG1* and *GsAG2* were categorized into the PLE/SHP subgroup, rather than the AG/FAR subgroup. In expression analyses, *GsAG1* transcripts were detected in the second to fourth floral whorls, while *GsAG2* transcripts were detected in only the inner two whorls. Transgenic Arabidopsis expressing *GsAG1* lacked petals and formed carpeloid organs instead of sepals. Compared with a single-flowered gentian cultivar, a double-flowered gentian mutant showed decreased expression of *GsAG1* but unchanged expression of *GsAG2*. An analysis of the genomic structure of *GsAG1* revealed that the gene had nine exons and eight introns, and that a 5,150-bp additional sequence was inserted into the sixth intron of *GsAG1* in the double-flowered mutant. This insert had typical features of a *Ty3*/*gypsy*-type LTR-retrotransposon, and was designated as *Tgs1*. Virus-induced gene silencing of *GsAG1* by the *Apple latent spherical virus* vector resulted in the conversion of the stamen to petaloid organs in early flowering transgenic gentian plants expressing an Arabidopsis *FT* gene.

**Conclusions:**

These results revealed that *GsAG1* plays a key role as a C-functional gene in stamen organ identity. The identification of the gene responsible for the double-flowered phenotype will be useful in further research on the floral morphogenesis of Japanese gentian.

**Electronic supplementary material:**

The online version of this article (doi:10.1186/s12870-015-0569-3) contains supplementary material, which is available to authorized users.

## Background

Double-flowered plants are often preferred by consumers because they are larger, more floriferous, and more showy than single flowers [1]. Double-flowered varieties are more common than single-floweredvarieties for several important floricultural plants including carnation (*Dianthus caryophyllus*), rose (*Rosa hybrida*), and chrysanthemum (*Chrysanthemum × morifolium*). In other floricultural plants, the development of double-flowered varieties is one of the main breeding aims alongside improvements to floral color, size, scent, vase life, and disease resistance.

Generally, the flowers of dicotyledonous plants are composed of four types of organs; sepals, petals, stamens, and pistils, which are arranged in four whorls. In eudicots, floral organ identities are explained by the ABC model, which has been established from studies on two model plants, *Arabidopsis thaliana* and *Antirrhinum majus* [2]. The ABC model includes many genes encoding MADS-box transcription factors. According to this model, there are three classes of gene functions. The A-function gene, *APETALA1* (*AP1*, *SQUAMOSA* (*SQUA*) in *A. majus*), is expressed in the first and second whorls. The B-function genes, *APETALA3* (*AP3, DEFICIENCE* (*DEF*) in *A. majus*) and *PISTILLATA* (*PI, GLOBOSA* (*GLO*) in *A. majus*) are expressed in the second and third whorls, and their encoded proteins gain their B-function when they form heterodimers [3]. The C-function genes are expressed in the third and fourth whorls, and play an important role in stamen and pistil formation. Male and female organ identities are specified by a single C-function gene, *AGAMOUS* (*AG*), in Arabidopsis, but by two C-function genes, *PLENA* (*PLE*) and *FARINELLI* (*FAR*), in *A. majus* [4]. The *A. majus ple* mutant was shown to form petal and petaloid organs in place of stamens and carpels, respectively [5], similar to the Arabidopsis *ag-1* mutant. *A. majus PLE* is an ortholog of Arabidopsis *SHATTERPROOF 1/2* (*SHP1/2*), which is involved in the dehiscence of mature fruit [6], but it is not an ortholog of AG. AG/FAR and SHP/PLE are paralogs, but not orthologs derived from a duplication event in a common ancestor [7].

To control floral organ identity, the B- and C-function genes also require *SEPALLATA* (*SEP*), which is defined as an E-function gene [8]. The proposed “quartet model” directly links floral organ identity to the action of four different tetrameric transcription factor complexes composed of MADS-box proteins [9, 10]. Petunia *FBP6* and *FBP11* are expressed in the ovule, and are defined as D-class *MADS-box* genes [11]. Recently, the petunia C- and D-clade genes were shown to have largely overlapping functions specifying ovule identity and floral termination [12]. D-function genes have also been identified in lily (*LMADS2*, [13]), *Eustoma grandiflorum* (*EgMADS2*, [13]), and Arabidopsis (*STK*, [14]).

The deficiency of C-function genes results in the conversion of third-whorl stamens to petals, and fourth-whorl pistils to sepals [15]. This sepal-petal-petal pattern repeats itself many times, resulting in flowers with many petals. In addition to its role in determining floral organ identity, AG also plays a role in terminating flower development [16, 17]. Double-flowered phenotypes result from C-function deficiency in most floricultural plants, including *Ipomoea nil* [18]*, Rosa hybrida* [19], *Petunia hybrida* [20], *Cyclamen persicum* [21], and *Cymbidium ensifolium* [22]. Therefore, it is likely that double-flowers of Japanese gentian plants result from lost or impaired C-function gene (s), although this had not been confirmed experimentally.

Japanese gentian (*Gentiana scabra*, *Gentiana triflora,* and their interspecific hybrids) is one of the most popular floricultural plants in Japan, and is used as cut flowers and potted plants [23]. The genus *Gentiana* comprises more than 400 species, and belongs to the family Gentianaceae, which also contains the genera *Eustoma*, *Swertia*, and *Tripterospermum*. The flowers of Japanese gentian have a bell-shaped corolla with five lobes, five stamens partly fused with petals, and one pistil. Organs known as plicae, which are located between the lobes of the corolla, are a typical feature of the *Gentiana* genus. The petals of Japanese gentians are vivid blue, which is conferred by the polyacylated anthocyanin gentiodelphin [24]. The flavonoids of Japanese gentian, the structures of the anthocyanins and flavones, and the biosynthetic structural and regulatory genes associated with these pigments have been well studied [25]. More recently, we determined the structures of flavones that accumulate in the leaves and flowers of *G. triflora* and identified a novel glucosyltransferase gene involved in the formation of flavone-glucosides [26].

However, there have been few studies on the floral morphogenesis in Japanese gentian at the molecular level. Floral homeotic *MADS-box* genes have been isolated and characterized from *E. grandiflorum*, which belongs to the family Gentianaceae [27]. Although Mishiba et al. [28] isolated four *MADS-box* genes from *G. triflora* (*GtMADS1*–*GtMADS4*; Genbank accession numbers AB189429–AB189432), these genes have not been characterized in detail. To date, there have been no systematic characterizations of floral morphological *MADS-box* genes in Japanese gentian.

Here, we attempted to characterize a double-flowered mutant of *G. scabra,* a species closely related to *G. triflora*. We isolated and characterized *MADS-box* genes expressed in gentian flower buds, focusing on C-class *MADS-box* genes. We identified 14 *MADS-box* genes belonging to A, B, C, D, and E classes; these genes are presumably involved in floral development and organ identification. Analyses of a double-flowered mutant revealed that the phenotype was caused by an insertion of a novel retrotransposable element (*Tgs1*) into one of the C-function genes, *GsAG1*. This was confirmed by suppressing *GsAG1* using the *Apple latent spherical virus* (ALSV) vector. To our knowledge, this is the first report of the functional characterization of *MADS-box* genes involved in the floral morphogenesis of Japanese gentian, and the involvement of a retrotransposable element in its double-flowered phenotype.

## Results

### Isolation of *MADS-box* genes from Japanese gentian

**Fig. 1:**
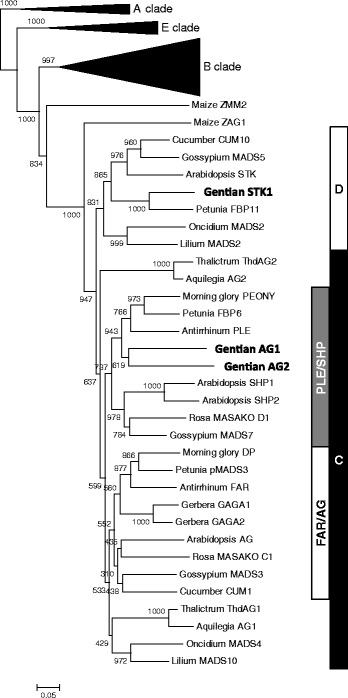
Phylogenetic tree of C/D-class MADS-box proteins. Phylogenetic tree was constructed by the neighbor-joining method using ClustalW and visualized using MEGA6. Genbank accession numbers of amino acid sequences used in phylogenetic analysis are as follows: *Arabidopsis thaliana* AG (NP_567569), SHP1 (NP_001190130), SHP2 (NP_850377) and STK (NP_192734); *Antirrhinum majus* FAR (CAB42988) and PLE (AAB25101); *Aquilegia alpina AG1*(AAS45699) and AG2 (AAS45698); *Cucumis sativus* CUM1 (AAC08528) and CUM10 (AAC08529); *Gentiana scabra* GsAG1 and GsAG2 (this study); *Gerbera hybrida* GAGA1 (CAA08800) and GAGA2 (CAA08801); *Gossypium hirsutum* MADS3 (AAL92522), MADS5 (ABM69043) and MADS7 (ABM69045); *Ipomoea nil* DP (BAC97837) and PEONY (BAC97838); *Lilium longiflorum* MADS2 (AAS01766) and MADS10 (AIJ29174); *Oncidium hybrida* MADS2 (AIJ29175) and MADS4 (AIJ29176); *Petunia hybrida* FBP6 (CAA48635), FBP11 (CAA57445), PFG (AAF19721) and pMADS3 (Q40885); *Rosa rugosa* MASAKO C1 (BAA90744) and MADSKO D1 (BAA90743); *Thalictrum dioicum* ThdAG1 (AAS45683) and ThdAG2 (AAS45682); *Zea mays* ZAG1 (AAA02933) and ZMM2 (NP_001104926). Numerals beside branches indicate bootstrap values from 1,000 replicates. Scale bar indicates 0.05 amino acid substitutions per site

The fragments of Japanese gentian *MADS-box* genes were amplified using degenerate primers designed from the conserved domain of AGAMOUS proteins, as described by Kramer et al. [29, 30]. After subcloning, 96 clones were sequenced, and 14 independent clones were identified. Using 5′-RACE technology, we obtained eight independent clones of complete full-length cDNA sequences, whereas the 5′-upstream fragments corresponding to the other six clones were not obtained. In a phylogenetic analysis based on the deduced amino acid sequences, these Japanese gentian *MADS-box* genes clustered into four functional clades (Fig. 1, Additional file 1: Figures S1 and S2).

There were two gentian A-clade *MADS-box* genes; *GsAP1* (Genbank accession number LC022772) and *GsFUL* (LC022780). Core eudicot species have two types of A-class MADS-box lineage genes, *euAP1* and *euFUL* [31]. *GsAP1* and *GsFUL* were categorized into *euAP1* and *euFUL*, respectively (Additional file 1: Figure S1). The deduced amino acid sequence of *GsAP1* showed 63.9 % identity with that of *GsFUL*.

We also identified another six *MADS-box* genes, which were categorized as B-class genes (Additional file 1: Figure S2). The B-class *MADS-box* genes form three subgroups, *euAP3/DEF*, *TM6* (paleoAP3), and *PI/GLO* [32]. *GsAP3a* (LC022769) and *GsAP3b* (LC022774) were categorized into the *AP3/DEF* subgroup, while *GsPI1* (LC022770), *GsPI2* (LC022771), and *GsPI3* (LC022773) were categorized into the *PI/GLO* subgroup. *GsTM6* (LC022767) belonged to the TM6 subgroup derived from the *AP3/DEF* subgroup. The deduced amino acid sequence of *GsAP3a* exhibited 78.0 % and 59.8 % identities with those of *GsAP3b* and *GsTM6*, respectively. The deduced amino acid sequence of GsAP3b showed 60.1 % identity with that of GsTM6. GsAP3a exhibited 60.3 %, 77.1 %, and 72.4 % identities, while GsAP3b exhibited 56.7 %, 71.5% and 73.1 % with Arabidopsis AP3, *Antirrhinum* DEF, and petunia GP, respectively. GsTM6 exhibited 58.8 %, 57.3 %, and 52.4 % identities with tomato TDR6, petunia TM6, and rose MADSKO B3, respectively. GsPI1 exhibited 93.7 % and 86.3 % identity with GsPI2 and GsPI3, respectively, while GsPI2 showed 80.2 % identity with GsPI3. The GsPIs exhibited 55.7 %–58.9 %, 58.1 %–64.2 %, 68.1 %–70.8 %, and 59.9 %–67.3 % identities with Arabidopsis PI, *Antirrhinum* GLO, petunia pMADS2, and petunia GLO1, respectively.

The C-clade *MADS-box* genes can be separated into two subgroups, *AG/FAR* and *SHP/PLE* [7]. We isolated two Arabidopsis *AG*/*SHP* orthologs, *GsAG1* (LC022775) and *GsAG2* (LC022779), from Japanese gentian floral buds, and both belonged to the *SHP/PLE* subgroup (Fig. 1). No clones in the AG/FAR subgroup were obtained by degenerate PCR or by searching the gentian flower normalized library described by Nakatsuka et al. [33]. The deduced amino acid sequence of *GsAG1* showed 63.9 % identity with that of *GsAG2*. GsAG1 showed 68.8 %, 66.8 %, and 65.2 % amino acid sequence identity with petunia FBP6 [34], *A. majus* PLENA [5], and *I. nil* PEONY [18], respectively, whereas GsAG2 showed 68.4 %, 63.5 %, and 66.4 % identity, respectively.

GsSTK1 (LC022768) showed high sequence similarity to STK (AGL11), which is encoded by a D-class *MADS-box* gene in Arabidopsis and regulates ovule development [35]. The deduced amino acid sequence of GsSTK1 showed 85.1 %, 80.9 %, and 64.9 % identity with that of *Eustoma grandiflorum* MADS1 [13], petunia FBP7 [36] and Arabidopsis STK [14], respectively. We also isolated three *SEP* orthologs, designated as *GsSEP1* (LC022776), *GsSEP2* (LC022777), and *GsSEP3* (LC022778), all of which were E-function *MADS-box* genes (Additional file 1: Figure S1).

 The A-function genes included AP1-like *MADS-box* genes, and also AP2-like genes harboring two continuous AP2 domains. We isolated a *GsAP2* ortholog (LC022781) from the gentian petal normalized library described by Nakatsuka et al. [33]. The *GsAP2* cDNA was 1,813-bp long, and encoded a protein of 456 amino acid residues (Additional file 1: Figure S3). The *miR172*-target nucleotide sequences of *AP2* were conserved within the *GsAP2* coding regions.

### Spatial expression analysis of *MADS-box* genes in different floral organ, leaves, and stems

**Fig. 2:**
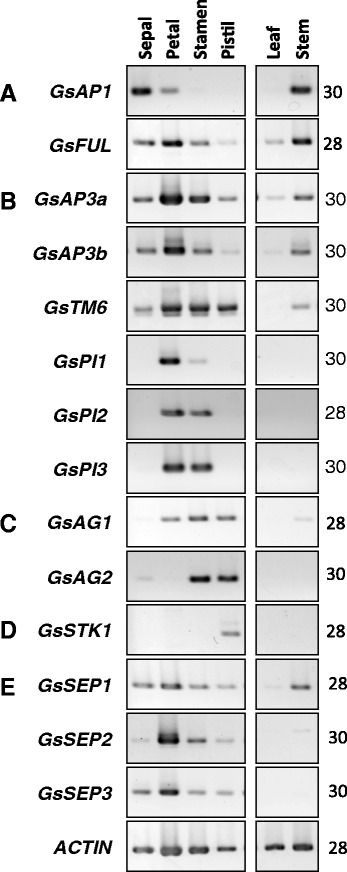
Spatial expression profiles of *MADS-box* genes in floral organs of Japanese gentian. Semi-quantitative RT-PCR analysis was performed using total RNAs isolated from sepals, petals, stamens, and pistils of floral buds, and from leaves and stems. Expression profiles of 14 MADS-box genes were investigated: A-clade (*GsAP1* and *GsFUL*), B-clade (*GsAP3a*, *GsAP3b*, *GsTM6*, *GsPI1*, *GsPI2* and *GsPI3*), C-clade (*GsAG1* and *GsAG2*), D-clade (GsSTK1), and E-clade (*GsSEP1*, *GsSEP2* and *GsSEP3*) genes. *Actin* served as the reference gene. Gene names and cycle numbers are indicated at the left and right of panel, respectively

The spatial expression patterns of isolated *MADS-box* genes were analyzed by semi-quantitative RT-PCR in wild-type Japanese gentian (Fig. 2). Among the A-clade *MADS-box* genes, *GsAP1* expression was restricted to the first and second whorls and stem tissues, while *GsFUL* transcripts were detected in all of the tissues tested. *GsFUL* was strongly expressed in the first and second floral whorls and also in stem tissues.

The expressions of *GsAP3a, GsAP3b,* and *GsTM6*, belonging to the AP3/DEF subfamily*,* were detected in all four whorls of the floral organs. There were high transcript levels of *GsAP3a* and *GsAP3b* in the petal and stamen, and high transcript levels of *GsTM6* in the pistil organs in addition to whorls 2 and 3. Transcripts of *GsAP3a*, *GsAP3b,* and *GsTM6* were detected in stem organs, but barely detected in leaves. In contrast to the AP3/DEF subfamily, the PI/GLO subfamily genes *GsPI1*, *GsPI2* and *GsPI3* were expressed only in the petal and stamen organs (Fig. 2). The transcript levels of *GsPI2* and *GsPI3* were approximately equal in the petal and stamen organs, whereas there were higher transcript levels of *GsPI1* in the petal than in the stamen. The three *GsPI* genes were expressed at undetectable levels in vegetative organs. Thus, the expression profiles of the *GsPI* genes belonging to PI/GLO subgroup differed from those of the genes in the AP3/DEF and TM6 subgroups.

The two C-class *MADS-box* genes, *GsAG1* and *GsAG2*, were strongly expressed in the third (stamen) and fourth whorls (pistil). Transcripts of *GsAG1* were also present in petals. Transcripts of both *GsAG1* and *GsAG2* were at very low levels or undetected in vegetative tissues (leaves and stems). Transcripts of *GsSTK1* were detected only in pistils, and not in other whorls, leaves, or stems. The three E-class *MADS-box* genes, *GsSEP1*, *GsSEP2,* and *GsSEP3*, showed similar expression profiles in floral organs. Transcripts of *GsSEP*2 and *GsSEP3* were detected all floral whorls but not in leaves or stems, whereas *GsSPE1* transcripts were detected in all floral whorls and in stems.

### Heterologous expressions of *GsAG1* and *GsAG2* in Arabidopsis

**Fig. 3:**
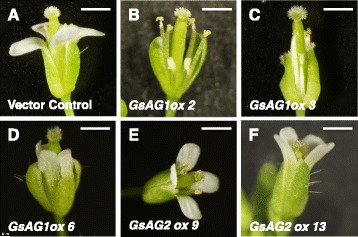
Typical floral phenotypes of *GsAG1-* and *GsAG2*-expressing transgenic Arabidopsis plants. **a** Vector-control flower with normal sepal and petal organs. **b**–**d** Flowers of *GsAG1*-overexpressing transgenic lines nos. 2, 3, and 6 with sepals and petals converted into pistiloid and stamenoid organs, respectively. **e**–**f** Flowers of *GsAG2*-overexpressing transgenic lines nos. 9 and 13 with normal floral phenotypes. Expression of transgene in each T_2_ transgenic plant is illustrated in Additional file 1: Figure S4. Bar = 10 mm

To investigate the functions of *GsAG1* and *GsAG2*, we produced four and six lines of T_2_ transgenic Arabidopsis plants overexpressing *GsAG1* or *GsAG2*, respectively. Ectopic expressions of C-class *MADS-box* genes in Arabidopsis and tobacco have been used to evaluate the function of AG orthologs from several plants [37, 38]. Ectopic expressions of *AG* genes have been shown to induce the *ap2* mutant phenotype; that is, pistil-stamen-stamen-pistil [39] Of the four *GsAG1-*overexpressing Arabidopsis lines, three formed carpeloid organs instead of sepals, and showed partial disappearance of petals (Fig. 3b–d). No morphological changes were observed in all six *GsAG2-*overexpressing Arabidopsis lines (Fig. 3e–f). These results revealed that the biological functional ortholog of Arabidopsis *AG* was *GsAG1,* not *GsAG2*.

### Expression analysis of *MADS-box* genes in a double-flowered mutant

**Fig. 4:**
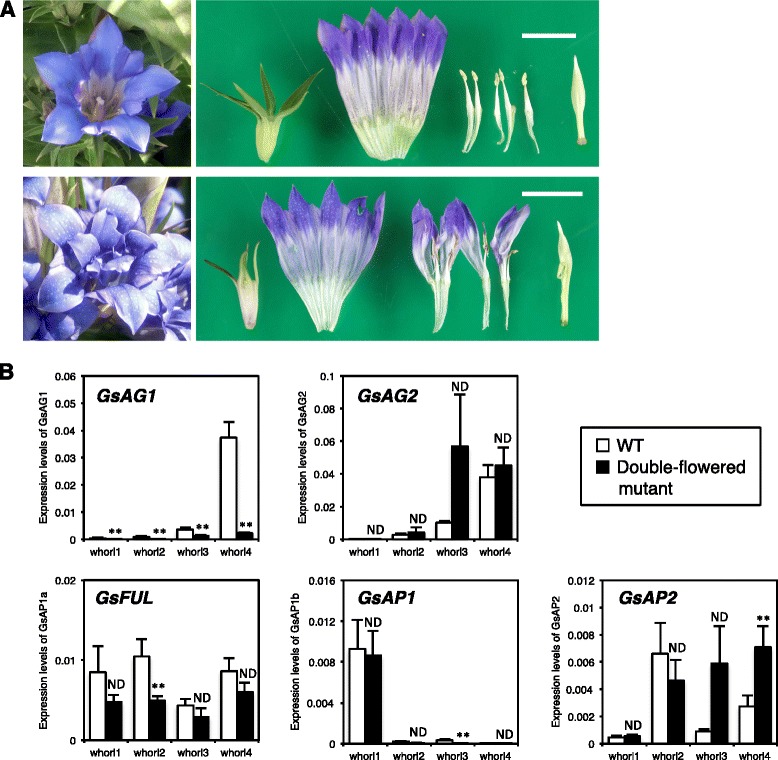
Phenotype of double-flowered gentian mutant and spatial expression analysis of *MADS-box* genes. **a** Typical floral phenotypes of single flower cv. Alta (upper panels) and double-flowered mutant (lower panels). Bar = 2 cm. **b** qRT-PCR analysis of floral *MADS-box* genes in single-flowered cultivar (WT) and double-flowered mutant. Total RNAs were isolated from each whorl organ of floral buds at flower developmental stage 3 as defined by Nakatsuka et al. [58]. Values are the average of four biological replicates ± standard deviation. White bar indicates single-flowered gentian cv. Alta. Black bar indicates double-flowered mutant. ** and ND indicate significant difference (*P* < 0.01) and no significant difference, respectively (Student’s *t*-test)

Next, we attempted to identify the cause of double-flowers in a gentian mutant. The double-flowered mutant had petaloid organs instead of stamens in the third whorl (Fig. 4a). The petaloid organ consisted of a petal structure fused to a sterile stamen. Some individuals of the double-flowered mutant also formed a slightly abnormal pistil that contained another incomplete pistil.

To identify the candidate gene responsible for the formation of double flowers, we compared the spatial expression profiles of C-class *MADS-box* genes between the double-flowered mutant and the typical single-flowered gentian cv. Alta (Fig. 4b). The transcript levels of *GsAG1* in the third and fourth whorls were significantly lower in the double-flowered mutant than in the single-flowered cultivar. In contrast, the abundance of *GsAG2* transcripts was not significantly different between the wild-type cultivar and the double-flowered mutant. The transcript levels of *GsAP2* in the inner two whorls were higher in the double-flowered mutant than in the wild-type plants (Fig. 4b). There were also differences between the wild-type cultivar and the double-flowered mutant in the transcription profiles of other A-class *GsAP1* and *GsFUL* genes in the second and third whorls. Slight differences in the expression patterns of some genes might be because of the different genetic backgrounds of the single-flowered cultivar and the double-flowered mutant. However, these results suggested that *GsAG1*, a C-class *MADS-box* gene, was the most likely candidate gene responsible for the double-flowered phenotype.

### Genomic structures of *GsAG1* and *GsAG2* in Japanese gentian

In spatial expression analyses of Japanese gentian *MADS-box* genes, reduced *GsAG1* transcript levels were detected in male and female organs of the double-flowered mutant (Fig. 4b). Therefore, we determined the genomic sequences of *GsAG1* and *GsAG2* in the double-flowered mutant and control plants.

**Fig. 5:**
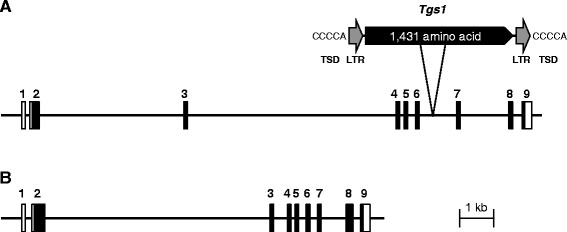
Genomic structures of *GsAG1* and *GsAG2.* **a** Genomic structure of *GsAG1* in Japanese gentian. Open boxes show untranslated regions, filled boxes show translated regions with exons. Numerals above boxes indicate exon number. Scale bar indicates 1 kb. Open arrow indicates insertion position of transposable element *Tgs1* in *gsag1* in double-flowered mutant. TSD, target site duplication; LTR, long terminal repeat. *Tgs1* is 5,150-bp long with an ORF encoding 1,431 amino acid sequences of a gag-pol polyprotein, 334 bp of LTRs, and 5 bp of TSDs. **b** Genomic structure of *GsAG2* in Japanese gentian cv. Alta

The genome sequence corresponding to *GsAG1* cDNA was 15.3-kb long, and consisted of nine exons and eight introns (Fig. 5a). The number and position of introns were conserved between Arabidopsis *AG* and *GsAG1*. The second and third introns of *GsAG1* (4.3 kb and 6.7 kb, respectively) were considerably longer than those of the corresponding introns in *AG* genes in other plants (2,998 bp and 102 bp, respectively, in Arabidopsis). The genomic sequence of *GsAG2* was 9.5-kb long and consisted of nine exons and eight introns, like *GsAG1* (Fig. 5b). The second intron of *GsAG2* was 6.6-kb long, but the third intron was shorter than that of *GsAG1.* The second intron of Arabidopsis *AG* contains transcriptional regulation regions [7, 40]. The second intron region of both *GsAG1* and *GsAG2* had several *cis*-elements; a CArG box (CW_8_G), a LFY binding site (CCANTG) and a 70-bp region (CCAATCA repeat) (data not shown).

### Genomic structures of *GsAG1* and *GsAG2* in the double-flowered mutant

 Next, we compared the genomic structures of *GsAG1* and *GsAG2* between the wild-type cultivar and the double-flowered mutant. Genomic PCR analyses targeting the sixth intron region of *GsAG1* amplified a fragment from wild type, but not from the double-flowered mutant (data not shown). Therefore, we sequenced the sixth intron of *GsAG1* in the double-flowered mutant using genome walking technology. The sixth intron of *GsAG1* in the double-flowered mutant had a 5,150-bp insertion that was not present in wild type. This inserted sequence had typical features of an LTR-retrotransposon, including a 5-bp target site duplication (TSD, CCCCA) and a 334-bp perfectly matching long terminal repeat (LTR) at both ends (Fig. 5a). The insert was designated as *Tgs1* (transposable element of *Gentiana scabra* 1). *Tgs1* encoded a 1,431-amino acid sequence of a *gag-pol* polyprotein belonging to the *Ty3*/*gypsy*-type retrotransposon group. There was no difference in the genomic structure of *GsAG2* between the double-flowered mutant and the wild-type cultivar (data not shown).

### Suppression of *GsAG1* by virus-induced gene silencing

 To confirm whether the deficiency of the *GsAG1* gene contributed to the double-flowered phenotype in Japanese gentian, we attempted to suppress the expression of *GsAG1* using VIGS. We used *Apple latent spherical virus* (ALSV) vectors because they have been used for reliable and effective VIGS in a broad range of plants [41, 42].

Gold particles coated with pEALSR1 and pEALSR2L5R5 were bombarded into *in vitro-*grown plants of transgenic Japanese gentian overexpressing *AtFT* [43]. One month after the bombardment, the proliferation of ALSV in inoculated plants was confirmed by RT-PCR analysis. The proliferation of ALSV was detected in almost all plantlets (data not shown), confirming that the direct bombardment of plasmid vectors was suitable to inoculate ALSV into gentian.

**Fig. 6:**
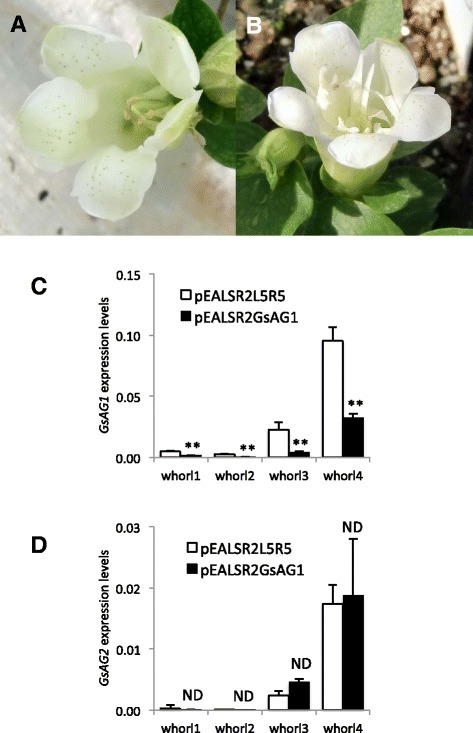
Effects of *GsAG1* suppression by VIGS. Typical flower phenotypes of control ALSV-empty (pEALSR1/pEALSR2L5R5, **a**) and ALSV-AG1 (pEALSR1/pEALSR2-GsAG1)-infected plants (**b**). Spatial expression patterns of *GsAG1* (**c**) and *GsAG2* (**d**) in ALSV-empty and ALSV-GsAG1-infected plants. Flowers from three independent plants were examined for each treatment. Values are mean ± standard deviation (*n* = 3). ** and ND indicate significant difference (*P* < 0.01) and no significant difference, respectively (Student’s *t*-test)

 Twenty-two and 20 *AtFT*-overexpressing gentian plants were inoculated with either an empty ALSV vector (pEALSR1/pEALSR2L5R5) or the ALSV-GsAG1 vector (pEALSR1/pEALSR2-GsAG1), respectively. RT-PCR analysis confirmed that the biolistic inoculation of ALSV vectors resulted in a 90 % inoculation frequency (data not shown). The gentian plants inoculated with ALSV vectors were acclimated in a closed greenhouse, and set flowers after 1–3 months of acclimation. There was no significant difference in flower phenotype between wild type and plants inoculated with an empty ALSV vector (Fig. 6a). Six out of 14 surviving plants inoculated with ALSV-GsAG1 formed petals in place of stamens (Fig. 6b). The qRT-PCR analysis showed that plants showing the conversion phenotype by infection with ALSV-GsAG1 had significantly suppressed *GsAG1* transcript levels, compared with those in plants inoculated with the empty vector (Fig. 6c). The transcript levels of *GsAG2* were not affected by ALSV-GsAG1 infection. There was no significant morphological change in the pistils of ALSV-GsAG1-inoculated plants.

## Discussion

In this study, we isolated 14 *MADS-box* genes expressed in floral buds of *G. scabra*: two A-class genes (*GsAP1* and *GsFUL*), six B-class genes (*GsAP1a*, *GsAP1b*, *GsTM6*, *GsPI1*, *GsPI2* and *GsPI3*), two C-class genes (*GsAG1* and *GsAG2*), one D-class genes (*GsSTK1*), and three E-class genes (*GsSEP1, GsSEP2,* and *GsSEP3*) (Fig. 1, Additional file 1: Figure S1 and Figure S2). Mishiba et al. [28] cloned four *MADS-box* genes, *GtMADS1*–*GsMADS4,* from *G. triflora*, a closely related species of *G. scabra*. Our analyses confirmed that GtMADS1–GtMADS4 are orthologs of *GsFUL*, *GsAG2*, *GsAG1,* and *GsSEP1*, respectively.

In Arabidopsis, AP1 and FUL function independently; the former controls sepal and petal identities, and the latter controls fruit development and determinacy [31]. In other core eudicots, plants with defective *AP1* genes formed leaf-like sepals, but their petal identity was unaffected [44]. Therefore, *euFUL* genes play an early role in promoting the transition to reproductive meristems and a late role in fruit development. In Japanese gentian, *GsAP1* expression was restricted to the first and second whorls of the floral bud and stem, and it was expressed strongly in the sepals and stems (Fig. 2). Conversely, *GsFUL* was expressed in all tested organs, and was strongly expressed in petals and stems (Fig. 2). As well as *GsAP1* and *FUL*, *GsAP2* might also act as an A-function gene (Additional file 1: Figure S1). *GsAP2* was strongly expressed in the second whorl (Fig. 4b). In Arabidopsis, the expression of *AP2* is regulated by miR172 through translational inhibition [45]. The nucleotide sequence of *GsAP2* contained a conserved *miR172* target sequence (data not shown). Therefore, the whorl-specific expression of *GsAP2* might be controlled by miR172 in gentian, like in other plants.

In Arabidopsis and *A. majus*, B-function, which specifies petal and stamen identities, is determined by a heterodimer consisting of one AP3/DEF protein and one PI/GLO protein [46, 47]. *AP3*/*DEF* lineages can be categorized into two subgroups; *euAP3* and *paleoAP3* [29]. *euAP3* is widely distributed in higher eudicots, whereas *paleoAP3* is distributed in lower eudicots, magnoliid dicots, monocots, and basal angiosperms [48]. In addition, a number of higher eudicot species contain both *euAP3* and *paleoAP3* (designated as *TM6*). *AP3/DEF* genes belonging to the euAP3 (*GsAP3a* and *GsAP3b*) and TM6 (*GsTM6*) groups were isolated from Japanese gentian (Additional file 1: Figure S2). Three *euAP3*, one *TM6*, and two *PI/GLO* genes were also identified from *Eustoma grandiflorum* in the family Gentianaceae [27]. Therefore, it seems that a *TM6* gene encoding a B-class MADS-box protein is present in the family Gentianaceae, but not in the Solanaceae [35] or Asteraceae [49]. Geuten and Irish [50] reported that the PI/GLO lineage was duplicated and separated into GLO1 and GLO2 lineages in the Solanaceae. Their results also implied that the GLO1 lineage has been lost from the Gentianales and the GLO2 lineage lost from the Lamiales. The results of the present study indicated GsPI1 to GsPI3 in Japanese gentian are in the GLO2 lineage (Additional file 1: Figure S2). *GsAP3a*, *GsAP3b*, and *GsTM6* were expressed in all floral whorls (Fig. 2). High transcript levels of *GsAP3a* and *GsAP3b* were detected in the second (petal) and third whorls (stamen), and GsTM6 was expressed at high levels in whorls 2–4. On the other hand, the expressions of the three *GsPIs* were clearly restricted to the second and third whorls (Fig. 2). These differences in expression profiles among *euAP3*, *TM6*, and *PI/GLO* were also reported in petunia [48]. In petunia, *PhTM6* is mainly expressed in third and fourth whorls and is involved in stamen development but not petal development, while *PhDEF* is involved in both petal and stamen development [51, 48].

Both *GsAG1* and *GsAG2* were categorized into the SHP/PLE subgroup but not the AG/FAR subfamily (Fig. 1). In this study, we could not find any paralogous genes belonging to the *AG/FAR* subgroup by degenerate PCR technology. In *E. grandiflorum*, which also belongs to the family Gentianaceae, three *SHP*/*PLE* subgroup genes (*EgPLE1* to *EgPLE3*) were identified, but no *AG*/*FAR* subgroup genes [27]. The *AG/FAR* subgroup of C-class *MADS-box* genes is responsible for male and female organ identity in several plant species. This subgroup of genes includes Arabidopsis *AG* [52], petunia *pMADS3* [53], and *I. nil DUPLICATED* (*DP*, [18]). Members of the SHP/PLE subgroup also play a major role in floral organ identity in *A. majus* [5]. Therefore, *AG/FAR* subgroup genes might have disappeared from some species in the Gentianaceae, leaving *SHP/PLE* subgroup genes to function as C-class genes, although further analysis such as whole-genome sequencing should be conducted to confirm this hypothesis.

There is only one C-class *MADS-box* gene, a single copy of *AG*, in Arabidopsis. However, there are two *AG* paralogs in some plant species, including *A. majus* (PLE/FAR, [4]), petunia (pMADS3/FBP6, [34]), cucumber (CUM1/CUM10, [34]), maize (ZAG1/ZMM2, [54]), *I. nil* (DP/IN, [18]), and cyclamen (CpAG1/CpAG2, [21]). In maize, *ZAG1* transcripts accumulate in developing ears rather than in tassels, whereas *ZMM2* transcripts are more abundant in tassels [54]. In the *ple* single mutant of *A. majus*, the fourth whorl develops as two sepaloid/carpeloid/petaloid organs. The fourth whorl organs of *ple*/*far* double mutants develop as four or five well-formed petals [4]. Thus, *PLE* and *FAR* appear to contribute unequally to the specification of male and female organs.

*GsAG1* transcripts were detected in the inner three whorls, whereas *GsAG2* transcripts were restricted to the third and fourth whorls (Fig. 2). *GsAG1* transcripts were detected in petal organs (whorl 2) in the RT-PCR analysis (Fig. 2) but not in the qRT-PCR analysis (Fig. 4). The RT-PCR and qRT-PCR analyses were performed using floral buds at different floral development stages, S1 (immature bud) and S3 (just before anthesis), respectively. In general, *AG* is expressed in either the third or fourth whorls [15]. Therefore, *GsAG1* expression in the second whorl in Japanese gentian appears to be a unique phenomenon. This may be because the petals and stamens of Japanese gentians are fused at their lower halves. Therefore, at an early floral developmental stage, young petal organs might contain stamen primordia. As shown in the qRT-PCR analysis (Fig. 4), no *GsAG1* transcripts were detected in the second whorl because both petal and stamen organs were completely distinguishable at the later stage of floral development.

The heterologous expression of *GsAG1* in transgenic Arabidopsis caused the conversion of sepals into carpeloid organs, indicating its *AG* function (Fig. 3b). In contrast, *GsAG2*-expressing Arabidopsis showed no significant changes in morphogenesis compared with the empty vector control (Fig. 3c). Ectopic expressions of Arabidopsis *AG* or *Antirrhinum PLE* specified homeotic conversion of the first and second whorl organs, causing sepals to develop as carpels and petals to develop as stamens [37, 7]. The ectopic expression of *Antirrhinum* FAR converted petals to stamens, but did not alter sepal identity [7]. Thus, heterologous expression analyses in Arabidopsis do not always correctly evaluate the function of C-class *MADS-box* genes from other plant species.

Most double-flowered phenotypes result from a deficiency of C-function genes [2]. The qRT-PCR analysis showed that *GsAG1* transcripts were markedly decreased in the third and fourth whorls of double-flowered Japanese gentian, compared with those in single-flowered wild-type Japanese gentian (Fig. 4b). No *GsAG1* transcripts were detected in the doubled-flower mutant by RT-PCR using several primer combinations (data not shown), and no truncated *GsAG1* transcripts were detected by 3′-RACE. A sequencing analysis revealed that the double-flowered mutant had an insertion of a 5,150-bp putative retrotransposable element in the sixth intron of *GsAG1* (Fig. 5a). This transposable element, *Tgs1*, had the typical features of *Ty3*/*gypsy*-type retrotransposable elements (Fig. 5a). In the *duplicated* (*dp*) mutant of *I. nil*, the mutation was due to the rearrangement of genomic structure by the *Em/Spm* transposable element [18]. The *Antirrhinum ple* mutant was shown to have an insertion of the *Tam3* transposable element in the second intron of *PLE*, and *ple* mRNA was hardly detected in the floral organs of the mutant [5]. It was also reported that a double-flowered ranunculid mutant was associated with the insertion of a solo LTR retrotransposon into the fourth exon of *ThAG1* [55]. Thus, it is likely that the expression of *GsAG1* would be interrupted by the insertion of the long transposable element in the sixth intron.

VIGS is a useful tool for the functional analysis of genes in horticultural plants that are recalcitrant to other means of genetic transformation [56]. Petunia plants in which both *pMADS3* and *FBP6* were silenced by VIGS formed petaloid organs in place of carpels, depending on the cultivar [57]. Most viral vectors are excluded from meristematic tissue, and therefore, gene silencing in the meristem is not possible in most instances [56]. In this study, we used VIGS to silence *GsAG1* and observed that stamens were converted into petaloid organs (Fig. 6b). These results strongly suggested that the deficiency of *GsAG1* was responsible for the double-flowered phenotype of this mutant. Enhanced transcript levels of *GsAP2* were detected in the third and fourth whorls of the double-flowered mutant (Fig. 4b). In contrast, the spatial expression profiles of *GsAP1* and *GsFUL* were similar between the single-flowered cultivar and double-flowered plants. Mizukami and Ma [39] reported that AG antagonizes the function of AP2. Therefore, we speculated that *GsAG1* controls the whorl-specific expression of *GsAP2*.

In the double-flowered gentian mutant, the fourth-whorl pistil was not converted into petals, possibly because of the function of GsAG2. Compared with single-flowered gentian, the double-flowered mutant showed increased expression of *GsAG2* in the third whorl (Fig. 4b). There were also increased transcript levels of *GsAG2* in double-flowered transgenic gentians in which *GsAG1* was suppressed by VIGS (Fig. 6b). In *Antirrhinum, PLE* is required for full expression of *FAR*, whereas *FAR* negatively regulates the expression of *PLE* [4]. It is possible that *GsAG1* negatively regulates the expression of *GsAG2* in the third whorl of Japanese gentian. Unfortunately, there are no *GsAG2*-deficient mutants in nature; therefore, to show the function of the GsAG2, the suppression of *GsAG2* by VIGS should be attempted in future studies. In cyclamen, *CpAG1* is involved in stamen formation, and the deficiency of *CpAG1* caused the homeotic conversion of stamens into petals, resulting in double-petal phenotypes [21]. Overexpression of CpAG2-SRDX (a chimeric repressor) in the cyclamen *cpag1* mutant resulted in a multiple-petal phenotype, and the conversion of pistils into petals [21]. Thus, two C-class MADS orthologs contribute to male and female organ identity. Noor et al. [57] demonstrated that VIGS suppression of both *MADS3* and *FBP6* resulted in the conversion of the stamen/carpel into petal/petaloid organs, resulting in double flowers.

The current hypothesis is that GsAG1 plays an important role in male organ identify, while GsAG2 plays important roles in female organ identity and in terminating flowering. To confirm this hypothesis, *GsAG2*- and *GsAG1/GsAG2*- knockdown or knockout lines of Japanese gentian should be generated and analyzed in further studies.

## Conclusions

We investigated the causal factor (s) of a double-flowered mutant in Japanese gentian. We isolated and characterized 14 *MADS-box* genes and revealed that a novel retrotransposable element (*Tgs1*) inserted into the sixth intron of *GsAG1* gene is responsible for the mutant flower phenotype. This was confirmed by ALSV-based VIGS system in combination with Arabidopsis *FT*-expressing early flowering transgenic gentian plants. Further investigations will be required to fully understand the developmental regulation of floral morphogenesis in Japanese gentian. As variation in floral shape is currently limited in Japanese gentians, we believe that this information will be helpful for breeding gentian cultivars with variation in floral shape in the future.

## Methods

### Plant materials

Japanese gentian (*Gentiana scabra*) cv. Alta was grown in a field at the Iwate Agricultural Research Center (Kitakami, Iwate, Japan). The double-flowered mutant was purchased from Iwasaki-Engai Co. (Kitahiroshima, Hokaido, Japan) and grown as potted plants in the greenhouse of Iwate Biotechnology Research Center. Floral bud samples were collected at developmental stage 1, as defined by Nakatsuka et al. [58], and then stored at −80 °C until RNA extraction.

### Isolation of MADS-box genes from gentian flower buds

Total RNAs were isolated from the floral buds of Japanese gentian and purified using RNAiso Plus and Fruit-mate kits (Takara-bio, Otsu, Shiga, Japan). The cDNAs were synthesized by an RNA PCR kit (AMV) Ver. 3 (Takara-bio). The candidate gentian *MADS-box* genes were isolated using degenerate primers as described by Kramer et al. [29, 30]. The amplified fragments were subcloned into the pCR4TOPO TA cloning vector (Invitrogen, Carlsbad, CA, USA) and sequenced using a BigDye terminator ver. 1.1 cycle sequencing kit and an ABI PRISM 3130xl DNA sequencer (Applied Biosystems, Foster City, CA, USA). To obtain the full-length cDNA of each gentian *MADS-box* gene, 5′-rapid amplification of cDNA ends (5′-RACE) was performed using a GeneRacer kit with SuperScript III RT (Invitrogen). The amplified fragments were subcloned and sequenced as described above. Nucleotide sequences were translated into deduced amino acid sequences using CLC Sequence Viewer 7 (CLC bio, Aarhus, Denmark) and compared using the BLAST network service at the National Center for Biotechnology Information. The phylogenetic tree was constructed using ClustalW with neighbor-joining algorithm and visualized using MEGA ver. 6 software [59].

The *GtAP2* ortholog, which is not categorized as a MADS-box protein, was found by BLAST searches of in-house gentian petal cDNA library data [33], and then the full-length cDNA was obtained using RACE technology as described above.

### Gene expression analysis

To investigate the spatial expression profiles of gentian *MADS-box* genes, we performed semi-quantitative reverse transcription-PCR (RT-PCR) and quantitative RT-PCR (qRT-PCR) analyses. Total RNAs (1 μg) were isolated from each organ as described above, and then genomic DNA was eliminated and cDNAs were synthesized using gDNA Eraser and PrimeScript RT, respectively (Takara-bio).

For the RT-PCR analyses, the reaction mixture (50 μL) consisted of 1 × *Ex Taq* buffer, 0.2 mM dNTPs, 0.4 μM each primer, 2.5 U *Ex Taq* polymerase (Takara-bio), and 1 μL template cDNA. The PCR cycling conditions were as follows: 2 min at 94 °C, 26–34 cycles of 20 s at 95 °C, 40 s at 55 °C, and 1 min at 72 °C, and final extension for 10 min at 72 °C. The PCR products were electrophoresed on a 1.5 % agarose gel in TAE buffer and then stained with ethidium bromide.

The qRT-PCR analyses were performed with the StepOne Plus system (Applied Biosystems) using SYBR GreenER qPCR Super Mix (Invitrogen) as described previously [33]. Briefly, the reaction mixture (10 μL) consisted of 1 × Master Mix, 0.2 μM each primer, and 1 μL template cDNA. The cycling conditions were as follows: 95 °C for 20 s, followed by 40 cycles of 95 °C for 1 s and 60 °C for 20 s. The specificity of each amplification reaction was checked by a melting curve analysis. Fluorescence was measured at the end of each annealing step. The data were analyzed using StepOne Plus Software Version 2.2.2. The transcript level each gene was calculated relative to that of the reference gene *GtUBQ*. qRT-PCR analyses were performed using four biological replicates, and data were statistically analyzed by Student’s *t*-test. The sequences of all primers used in this study are listed in Additional file 1: Table S1.

### Production of transgenic Arabidopsis plants

*GsAG1* and *GsAG2* ORFs under the control of the CaMV35S promoter were each inserted into a binary vector harboring the kanamycin resistance (*NPTII*) gene to produce the plasmids pSkan-35S:: GsAG1 and pSkan-35S:: GsAG2, respectively. Each binary vector was transformed into *Agrobacterium tumefaciens* EHA101 by electroporation (MicroPulser: Bio-Rad, Tokyo, Japan). *A. thaliana* ecotype col-1 was transformed by the floral dip method as described by Clough and Bent [60]. Positive transformants were selected on germination medium containing 50 mg L^−1^ kanamycin. *GsAG1*- and *GsAG2*-expressing T_2_ transgenic plants (homozygous) were obtained by self-pollination. The floral morphogenetic phenotypes were observed in four *GsAG1*-expressing lines and in six *GsAG2-*expressing lines.

### Determination of genomic structures of *GsAG1* and *GsAG2*

The genomic nucleotide sequences of *GsAG1* and *GsAG2* were obtained using genome walking technology with a GenomeWalker Kit (Clontech, Takara-bio). Genomic DNA was isolated from young leaves of *G. scabra* ‘Alta’ and the double-flowered mutant using Nucleon PhytoPure (GE Healthcare Ltd., Buckinghamshire, UK). Amplified fragments were subcloned and then sequenced as described above.

### Virus-induced gene silencing of *GsAG1* in gentian plants

To investigate the function of *GsAG1*, we conducted virus-induced gene silencing (VIGS) using the *Apple latent spherical virus* (ALSV) vector [41]. The trigger fragment was amplified using primers harboring *Xho*I or *Bam*HI sites (Additional file 1: Table S2), and then subcloned into the pGEM-T Easy vector (Promega, Madison, MI, USA). The fragment was excised by double-digestion with *Xho*I and *Bam*HI, and then ligated into pEALSR2L5R5 [61] digested with the same enzymes. Large-scale plasmid purification was conducted using a NuleoBond Xtra Midi plus kit (Macherey-Nagel, Takara-bio).

The ALSV vector was inoculated into *G. hybrida* ‘Polarno White’ plants overexpressing the Arabidopsis *FLOWERING LOCUS T* (*AtFT*) gene. The *AtFT*-expressing gentian plants flower earlier than wild-type gentian [43]; therefore, they are useful for studies on floral morphogenesis. Gentian plants expressing *AtFT* were grown *in vitro* and inoculated with ALSV vectors by the PDS-1000/He particle Delivery system (Bio-Rad Laboratory). A 0.5-mg aliquot of gold particles (1.0 μm diameter; Bio-Rad Laboratories, Hercules, CA, USA) was mixed with 100 μL plasmid solution, which contained 5 μg pEALSR1 and 5 μg pEALSR2L5R5 derivatives, 10 μL 10 M ammonium acetate, and 220 μL isopropanol. The mixture was kept at −20°C for at least 1 h. Gold particles coated with plasmid DNA were washed three times with 1 mL ethanol and re-suspended in 10 μL ethanol. Particles were bombarded with 1,100 psi pressure at a distance of 10 cm from the microcarrier holder. After bombardment, virus-infected plants were acclimated and then grown in a closed greenhouse until flowering.

### Availability of supporting data

The GenBank/EMBL accession numbers of genes identified in this study are: *GsAP1* (LC022772), *GsFUL* (LC022780), *GsAG1* (LC022775), *GsAG2* (LC022779), *GsSTK1* (LC022768), *GsSEP1* (LC022776), *GsSEP2* (LC022777), *GsSEP3* (LC022778) and *GsAP2* (LC022781).

Phylogenetic data have been deposited in TreeBASE respository and is available under the URL http://purl.org/phylo/treebase/phylows/study/TB2:S17877.

## Additional file

Additional file 1:
**Table S1.** Sequences of primers used for RT-PCR and qRT-PCR analyses. **Table S2.** Sequences of primers used for ALSV vector construction. **Figure S1.** Phylogenetic tree of A- and E-class MADS-box proteins. *Arabidopsis thaliana*, AP1 (NP_177074), CAL (NP_564243), FUL (NP_568929), SEP1 (NP_568322), SEP2 (AAU82009), SEP3 (NP_564214), SEP4 (NP_178466); *Antirrhinum majus* SQUA (CAA45228); *Gentiana scabra* GsAP1 and GsFUL (this study); *Petunia hybrida* FBP2 (Q03489) and FBP5 (AAK21248). Phylogenetic tree was constructed using the neighbor-joining method with ClustalW and visualized using MEGA6. Numerals beside branches indicate bootstrap values from 1,000 replicates. Scale bar indicates 0.05 amino acid substitutions per site. **Figure S2.** Phylogenetic tree of B-class MADS-box proteins. Phylogenetic tree was constructed by the neighbor-joining method using ClustalW and visualized using MEGA6. Genbank accession numbers of amino acid sequences used in phylogenetic analysis are as follows: *Antirrhinum majus* DEF (P23706), GLO (Q03378); *Arabidopsis thaliana*, AP3 (AAD51903), PI (AAD51984); *Chrysanthemum* × *morifolium* CDM86 (AA022986), CDM115 (AA022985); *Gentiana scabra* AP3a, AP3b, PI1, PI2, PI3, TM6 (this study); *Gerbera hybrida* GDEF1 (Q9ZS28), GDEF2 (Q9ZS27), GDEF3 (ACV53813), GGLO1 (Q9ZS26); *Onchidium hybrida* MADS3 (AA045824), MADS5 (ADJ67234), MADS8 (ADJ67236), MADS9 (ADJ67235); *Oryza sativa* MADS2 (AAB52709), MADS16 (Q944S9); *Petunia* × *hybrida* GLO1 (AAS46018), GP (CAA49567), pMADS2 (QO7474); *Phalaenopsis equestris* MADS2 (AAR26628), MADS3 (AAR26629), MADS4 (AAR26626), MADS5 (AAR26627), MADS6 (AAV83997); *Rosa rugosa* MASAKO B3 (BAB63261), MASAKO BP (BAB11939), MASAKO euB3 (BAC79180); *Solanum lycopersicum* TDR6 (XP_004232453); *Torenia fournieri* DEF (BAG24492), GLO (BAG24493). Numerals beside branches indicate bootstrap values from 1,000 replicates. Scale bar indicates 0.05 amino acid substitutions per site. **Figure S3.** Phylogenic tree constructed using deduced amino acid sequences of GsAP2 and other AP2 orthologs. Phylogenetic tree was constructed by the neighbor-joining method using ClustalW and visualized using MEGA6. Numerals beside branches indicate bootstrap values from 1,000 replicates. Amino acid sequences of AP2 orthologs were used as data sets as described by Karlova et al. [62]. Scale bar indicates 0.05 amino acid substitutions per site. **Figure S4.** Expression of transgenes in GsAG1-expressing and GsAG2-expressing T_2_ Arabidopsis plants. Four *GsAG1*-expressing lines (nos. 2, 3, 5 and 6) and six *GsAG2*-expressing lines (nos. 1, 3, 6, 7, 9 and 13) of transgenic plants were obtained. VC, vector control plants (harboring binary vector pIG121Hm). Semi-quantitative RT-PCR analysis was performed using total RNAs isolated from leaves. Analyses targeted *GsAG1*, *GsAG2,* and *Actin2* as a reference gene. Gene names and cycle numbers are indicated at the left and right of panel, respectively.

## Abbreviations

AG: AGAMOUS
ALSV: *Apple latent spherical virus*
AP1: APETALA1
AP2: APETALA2
FAR: FARINELLI
GLO: GLOBOSA
LTR: Long terminal repeat
PLE: PLENA
qRT-PCR: quantitative reverse transcription-PCR
RT-PCR: semi-quantitative reverse transcription-PCR
SEP: SEPALLATA
SHP: SHATTERPROOF
STK: SEEDSTICK
TSD: Target site duplication
VIGS: Virus-induced gene silencing

## References

[1] Reynold J, Tampion J (1983). Double flowers.

[2] Coen ES, Meyerowitz EM (1991). The war of the whorls: genetic interactions controlling flower development. Nature.

[3] McGonigle B, Bouhidel K, Irish VF. Nuclear localization of the Arabidopsis *APETALA3* and *PISTILLATA* homeotic gene products depends on their simultaneous expression. Genes Dev. 1996;10(14):1812–21. 10.1101/gad.10.14.1812 8698240

[4] Davies B, Motte P, Keck E, Saedler H, Sommer H, Schwarz-Sommer Z. PLENA and FARINELLI: redundancy and regulatory interactions between two *Antirrhinum* MADS-box factors controlling flower development. EMBO J. 1999;18(14):4023–34. 10.1093/emboj/18.14.4023 PMC1171478 10406807

[5] Bradley D, Carpenter R, Sommer H, Hartley N, Coen E. Complementary floral homeotic phenotypes result from opposite orientations of a transposon at the plena locus of *Antirrhinum*. Cell. 1993;72(1):85–95. 10.1016/0092-8674(93)90052-r 8093684

[6] Liljegren SJ, Ditta GS, Eshed Y, Savidge B, Bowman JL, Yanofsky MF. *SHATTERPROOF* MADS-box genes control seed dispersal in Arabidopsis. Nature. 2000;404(6779):766–70. 10.1038/35008089 10783890

[7] Causier B, Castillo R, Zhou J, Ingram R, Xue Y, Schwarz-Sommer Z (2005). Evolution in action: following function in duplicated floral homeotic genes. Curr Biol.

[8] Pelaz S, Ditta GS, Baumann E, Wisman E, Yanofsky MF. B and C floral organ identity functions require *SEPALLATA* MADS-box genes. Nature. 2000;405(6783):200–3. 10.1038/35012103 10821278

[9] Egea-Cortines M, Saedler H, Sommer H. Ternary complex formation between the MADS-box proteins SQUAMOSA, DEFICIENS and GLOBOSA is involved in the control of floral architecture in *Antirrhinum**majus*. EMBO J. 1999;18(19):5370–9. 10.1093/emboj/18.19.5370 PMC1171606 10508169

[10] Honma T, Goto K (2001). Complexes of MADS-box proteins are sufficient to convert leaves into floral organs. Nature.

[11] Colombo L, Franken J, Koetje E, van Went J, Dons HJ, Angenent GC, et al. The petunia MADS box gene *FBP11* determines ovule identity. Plant Cell. 1995;7(11):1859–68. 10.1105/tpc.7.11.1859 PMC161044 8535139

[12] Heijmans K, Ament K, Rijpkema AS, Zethof J, Wolters-Arts M, Gerats T (2012). Redefining C and D in the petunia ABC. Plant Cell.

[13] Tzeng TY, Chen HY, Yang CH (2002). Ectopic expression of carpel-specific MADS box genes from lily and lisianthus causes similar homeotic conversion of sepal and petal in Arabidopsis. Plant Physiol.

[14] Pinyopich A, Ditta GS, Savidge B, Liljegren SJ, Baumann E, Wisman E (2003). Assessing the redundancy of MADS-box genes during carpel and ovule development. Nature.

[15] Yanofsky MF, Ma H, Bowman JL, Drews GN, Feldmann KA, Meyerowitz EM (1990). The protein encoded by the Arabidopsis homeotic gene agamous resembles transcription factors. Nature.

[16] Lenhard M, Bohnert A, Jürgens G, Laux T (2001). Termination of stem cell maintenance in Arabidopsis floral meristems by interactions between WUSCHEL and AGAMOUS. Cell.

[17] Lohmann JU, Hong RL, Hobe M, Busch MA, Parcy F, Simon R (2001). A molecular link between stem cell regulation and floral patterning in Arabidopsis. Cell.

[18] Nitasaka E (2003). Insertion of an En/Spm-related transposable element into a floral homeotic gene DUPLICATED causes a double flower phenotype in the Japanese morning glory. Plant J.

[19] Dubois A, Raymond O, Maene M, Baudino S, Langlade NB, Boltz V (2010). Tinkering with the C-function: a molecular frame for the selection of double flowers in cultivated roses. PLoS One.

[20] Kapoor M, Baba A, Kubo K, Shibuya K, Matsui K, Tanaka Y, et al. Transgene-triggered, epigenetically regulated ectopic expression of a flower homeotic gene *pMADS3* in Petunia. Plant J. 2005;43(5):649–61. 10.1111/j.1365-313X.2005.02481.x 16115063

[21] Tanaka Y, Oshima Y, Yamamura T, Sugiyama M, Mitsuda N, Ohtsubo N (2013). Multi-petal cyclamen flowers produced by AGAMOUS chimeric repressor expression. Sci Rep.

[22] Wang SY, Lee PF, Lee YI, Hsiao YY, Chen YY, Pan ZJ, et al. Duplicated C-class MADS-box genes reveal distinct roles in gynostemium development in *Cymbidium**ensifolium* (Orchidaceae). Plant Cell Physiol. 2011;52(3):563–77. 10.1093/pcp/pcr015 21278368

[23] Nishihara M, Nakatsuka T, Mizutani-Fukuchi M, Tanaka Y, Yamamura S, da Silva J (2008). Gentian: from gene isolating to molecular breeding. Floriculture, ornamental and plant biotechnology.

[24] Goto T, Kondo T, Tamura H, Imagawa H (1982). Structure of gentiodelphin, an acylated anthocyanin isolated from, that is stable in dilute aqueous solution. Tetrahedron Lett.

[25] Nakatsuka T, Sasaki N, Nishihara M (2014). Transcriptional regulators of flavonoid biosynthesis and their application to flower color modification in Japanese gentians. Plant Biotechnol.

[26] Sasaki N, Nishizaki Y, Yamada E, Tatsuzawa F, Nakatsuka T, Takahashi H, et al. Identification of the glucosyltransferase that mediates direct flavone *C*-glucosylation in *Gentiana**triflora*. FEBS Lett. 2015;589(1):182–7. 10.1016/j.febslet.2014.11.045 25479084

[27] Ishimori M, Kawabara S (2014). Conservation and diversification of floral homeotic MADS-box genes in *Eustoma grandiflorum*. J Japanese Society Horticultural Sci.

[28] Mishiba K, Nishihara M, Nakatsuka T, Abe Y, Hirano H, Yokoi T (2005). Consistent transcriptional silencing of 35S-driven transgenes in gentian. Plant J.

[29] Kramer EM, Dorit RL, Irish VF. Molecular evolution of genes controlling petal and stamen development: duplication and divergence within the *APETALA3* and *PISTILLATA* MADS-box gene lineages. Genetics. 1998;149(2):765–83. 10.1093/genetics/149.2.765 PMC1460198 9611190

[30] Kramer EM, Jaramillo MA, Di Stilio VS (2004). Patterns of gene duplication and functional evolution during the diversification of the AGAMOUS subfamily of MADS box genes in angiosperms. Genetics.

[31] Litt A, Irish VF. Duplication and diversification in the* APETALA1*/*FRUITFULL* floral homeotic gene lineage: implications for the evolution of floral development. Genetics. 2003;165(2):821–33. 10.1093/genetics/165.2.821 PMC1462802 14573491

[32] Kramer EM, Irish VF (2000). Evolution of the petal and stamen developmental programs: Evidence from comparative studies of the lower eudicots and basal angiosperms. Int J Plant Sci.

[33] Nakatsuka T, Yamada E, Saito M, Fujita K, Nishihara M (2013). Heterologous expression of gentian MYB1R transcription factors suppresses anthocyanin pigmentation in tobacco flowers. Plant Cell Rep.

[34] Kater MM, Colombo L, Franken J, Busscher M, Masiero S, Van Lookeren Campagne MM (1998). Multiple AGAMOUS homologs from cucumber and petunia differ in their ability to induce reproductive organ fate. Plant Cell.

[35] Rounsley SD, Ditta GS, Yanofsky MF (1995). Diverse roles for MADS box genes in Arabidopsis development. Plant Cell.

[36] Angenent GC, Franken J, Busscher M, van Dijken A, van Went JL, Dons HJ (1995). A novel class of MADS box genes is involved in ovule development in petunia. Plant Cell.

[37] Airoldi CA, Bergonzi S, Davies B (2010). Single amino acid change alters the ability to specify male or female organ identity. Proc Natl Acad Sci U S A.

[38] Mandel MA, Gustafson-Brown C, Savidge B, Yanofsky MF (1992). Molecular characterization of the Arabidopsis floral homeotic gene APETALA1. Nature.

[39] Mizukami Y, Ma H (1992). Ectopic expression of the floral homeotic gene AGAMOUS in transgenic Arabidopsis plants alters floral organ identity. Cell.

[40] Deyholos MK, Sieburth LE. Separable whorl-specific expression and negative regulation by enhancer elements within the *AGAMOUS* second intron. Plant Cell. 2000;12(10):1799–810. 10.1105/tpc.12.10.1799 PMC149120 11041877

[41] Sasaki S, Yamagishi N, Yoshikawa N (2011). Efficient virus-induced gene silencing in apple, pear and Japanese pear using Apple latent spherical virus vectors. Plant Methods.

[42] Yamagishi N, Yoshikawa N (2013). Highly efficient virus-induced gene silencing in apple and soybean by apple latent spherical virus vector and biolistic inoculation. Methods Mol Biol.

[43] Nakatsuka T, Abe Y, Kakizaki Y, Kubota A, Shimada N (2009). M N. Over-expression of *Arabidopsis FT* gene reduces juvenile phase and induces early fowering in ornamental gentian plants. Euphytica.

[44] Pabón-Mora N, Ambrose BA, Litt A (2012). Poppy APETALA1/FRUITFULL orthologs control flowering time, branching, perianth identity, and fruit development. Plant Physiol.

[45] Chen XM (2004). A microRNA as a translational repressor of APETALA2 in Arabidopsis flower development. Science.

[46] Davies B, Egea-Cortines M, de Andrade SE, Saedler H, Sommer H (1996). Multiple interactions amongst floral homeotic MADS box proteins. EMBO J.

[47] Krizek BA, Meyerowitz EM (1996). Mapping the protein regions responsible for the functional specificities of the Arabidopsis MADS domain organ-identity proteins. Proc Natl Acad Sci U S A.

[48] Vandenbussche M, Zethof J, Royaert S, Weterings K, Gerats T. The duplicated B-class heterodimer model: whorl-specific effects and complex genetic interactions in *Petunia**hybrida* flower development. Plant Cell. 2004;16(3):741–54. 10.1105/tpc.019166 PMC385285 14973163

[49] Broholm SK, Pollanen E, Ruokolainen S, Tahtiharju S, Kotilainen M, Albert VA, et al. Functional characterization of B class MADS-box transcription factors in *Gerbera**hybrida*. J Exp Bot. 2010;61(1):75–85. 10.1093/jxb/erp279 PMC2791112 19767305

[50] Geuten K, Irish V (2010). Hidden variability of floral homeotic B genes in Solanaceae provides a molecular basis for the evolution of novel functions. Plant Cell.

[51] Rijpkema AS, Royaert S, Zethof J, van der Weerden G, Gerats T, Vandenbussche M. Analysis of the *Petunia**TM6* MADS box gene reveals functional divergence within the DEF/AP3 lineage. Plant Cell. 2006;18(8):1819–32. 10.1105/tpc.106.042937 PMC1533978 16844905

[52] Bowman JL, Smyth DR, Meyerowitz EM. Genetic interactions among floral homeotic genes of Arabidopsis. Dev. 1991;112(1):1–20. 10.1242/dev.112.1.1 1685111

[53] Tsuchimoto S, van der Krol AR, Chua NH. Ectopic expression of *pMADS3* in transgenic petunia phenocopies the petunia *blind* mutant. Plant Cell. 1993;5(8):843–53. 10.1105/tpc.5.8.843 PMC160320 8104573

[54] Mena M, Ambrose BA, Meeley RB, Briggs SP, Yanofsky MF, Schmidt RJ (1996). Diversification of C-function activity in maize flower development. Science.

[55] Galimba KD, Tolkin TR, Sullivan AM, Melzer R, Theissen G, Di Stilio VS (2012). Loss of deeply conserved C-class floral homeotic gene function and C- and E-class protein interaction in a double-flowered ranunculid mutant. P Natl Acad Sci USA.

[56] Senthil-Kumar M, Mysore KS (2011). New dimensions for VIGS in plant functional genomics. Trends Plant Sci.

[57] Noor S, Ushijima K, Murata A, Yoshida K, Tanabe M, Tanigawa T (2014). Double flower formation induced by silencing of C-class MADS-box genes and its variation among petunia cultivars. Sci Hortic.

[58] Nakatsuka T, Nishihara M, Mishiba K, Yamamura S (2005). Temporal expression of flavonoid biosynthesis-related genes regulates flower pigmentation in gentian plants. Plant Sci.

[59] Tamura K, Stecher G, Peterson D, Filipski A, Kumar S (2013). MEGA6: Molecular Evolutionary Genetics Analysis version 6.0. Mol Biol Evol.

[60] Clough SJ, Bent AF. Floral dip: a simplified method for *Agrobacterium*-mediated transformation of *Arabidopsis**thaliana*. Plant J. 1998;16(6):735–43. 10.1046/j.1365-313x.1998.00343.x 10069079

[61] Li C, Sasaki N, Isogai M, Yoshikawa N (2004). Stable expression of foreign proteins in herbaceous and apple plants using Apple latent spherical virus RNA2 vectors. Arch Virol.

[62] Karlova R, Rosin FM, Busscher-Lange J, Parapunova V, Do PT, Fernie AR (2011). Transcriptome and metabolite profiling show that APETALA2a is a major regulator of tomato fruit ripening. Plant Cell.

