# Supplementary material for: Isolation and characterization of the C-class MADS-box gene involved in the formation of double flowers in Japanese gentian
Source: BMC Plant Biol. 2015 Jul 17;15:182. doi: 10.1186/s12870-015-0569-3 (PMC4504037; doi:10.1186/s12870-015-0569-3)

Supplementary Table S1. Sequences of primers used for RT-PCR and qRT-PCR analyses.

Targeted genes	Primer sequence (5′ →3′) Fowerd	Reverse	Fragment length
For RT-PCR analysis			
GsAP1	AGCTCATGAGATTTCAGTTCTG	GCGCGGTATCAAGTTGTTGCTC	292
GsFUL	TCTCTGTGCTTTGTGATGCTGA	GAAGCGGTGGTTGAATAGATAG	539
GsTM6	ACACCAGTCCTACCACCACGAC	ACACCATCTCCGTCAACAAACG	420
GsAP3a	AATACATCAGCCCCGCCACTAC	TATGAGCTCTGGTTGCTACTCG	505
GsAP3b	AGCTGACTGTTCTTTGTGATGC	AGGTCCACCTCCATTTGAAAAC	474
GsPI1	GGATTGAAAACACAAGCAACAG	TAGGGCATCCTCTAAAACCATC	334
GsPI2	TGGATCAATACCAGCAGTCTAC	TCCATTCTCCACCACTCCTTTC	310
GsPI3	AGGAGTACATTAGCCCATCAAC	GTAGAGACGCCTTTTTCAAGAG	216
GsAG1	ATTGTCTTCTCAACCCGTGGTC	CCTGGCGAGAATAATGCTGATT	537
GsAG2	CTAGCAGTGGTCGCCTTTATGA	CAATCTCAGCAGCCAACAACTC	284
GsSTK1	GATGCTGAAGTTGCTCTTGTTG	TCATTGTATTCTTGCCCAGGTG	433
GsSEP1	TCTGTTCTCTGTGATGCTGAGG	AGGTGCTTGTTGTCTGCCTTCC	446
GsSEP2	CTCTGCAAGAGAAGCACTGGAG	CTGTAGTCTGGTATCCCATTTG	409
GsSEP3	TCTGTTCTCTGTGATGCTGAGG	ATGAGGATTCGGATTCCATTGG	437
GsActin2	CTAAGCAAAGCCAGCAAGTCCT	CACCAGAATCCAGCACAATACC	505
AtActin2			
For qRT-PCR analysis			
GsAP1	GCATGGAGAATATCCTGGAGAG	TCTTTGCAGCAACTCAACCTTA	137
GsFUL	GATCAATGGCACTGAATCTCAA	GGGGATTCAGATCTTCTCCTTC	122
GsAP2	TTCTGGGAAGCAAGTTTATCTAGG	TCAGCTATGGTGAAGTTGATGTCT	120
GsAG1	GTGCAGATAGCTGAAAATGAGAGA	AAGCCATGGATTGATGATGATT	97
GsAG2	GGAAGGAAGGGTTGAGAAAGGA	GAGCTCAATCTCAGCAGCCAA	76
Ubiquitin	CGATTGATTTTGTGAAGCCAAAA	TCCGGCAAATATCAACCTCTG	77

Supplementary Table S2. Sequences of primers used for ALSV vector construction.

Targeted genes	Primer sequence (5′ →3′)
GsAG1-U370(XhoI)	ctcgagAACAGGAACATGCTTGGGGAAG
GsAG1-L600(BamHI)	ggatccGGTGGCTCTCTCATTTTCAGC
GsAG2-U424(XhoI)	ctcgagTTGGAAGGAAGGGTTGAGAAAGG
GsAG2-L627(BamHI)	ggatccGTAATCAGATGCTGGCATGAA

Supplementary Figure S1

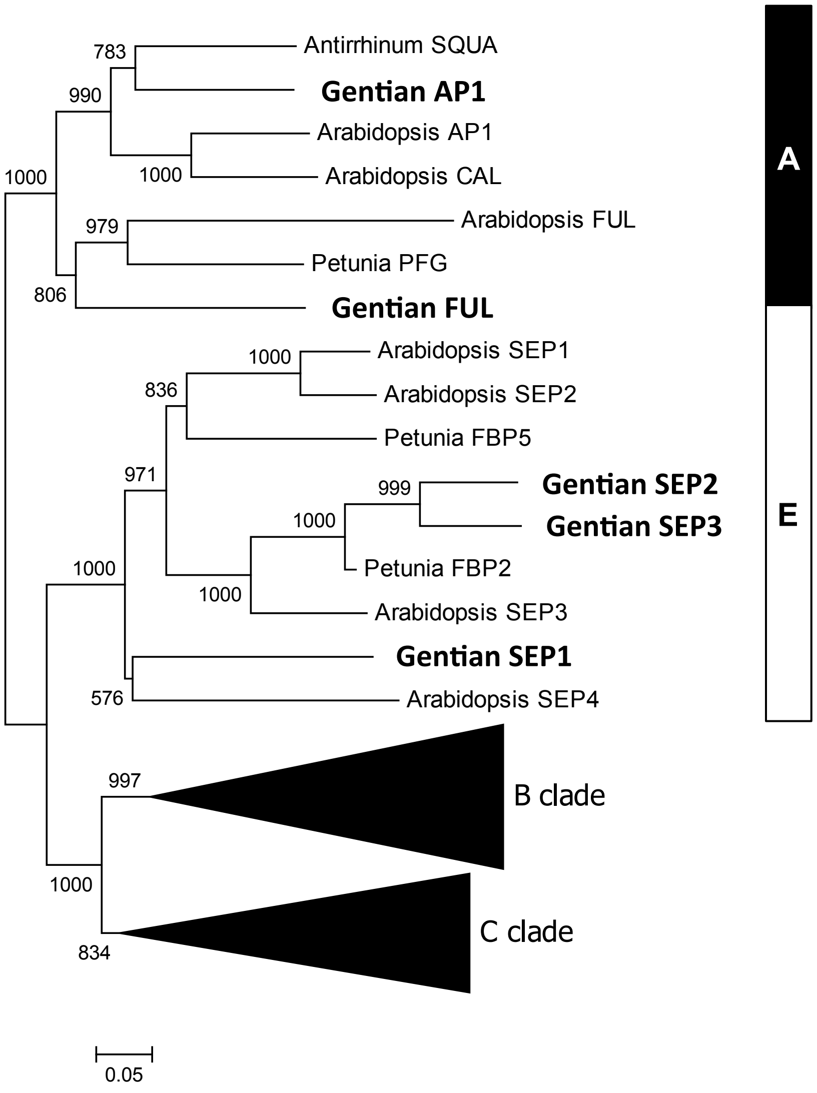

Supplementary Figure S2

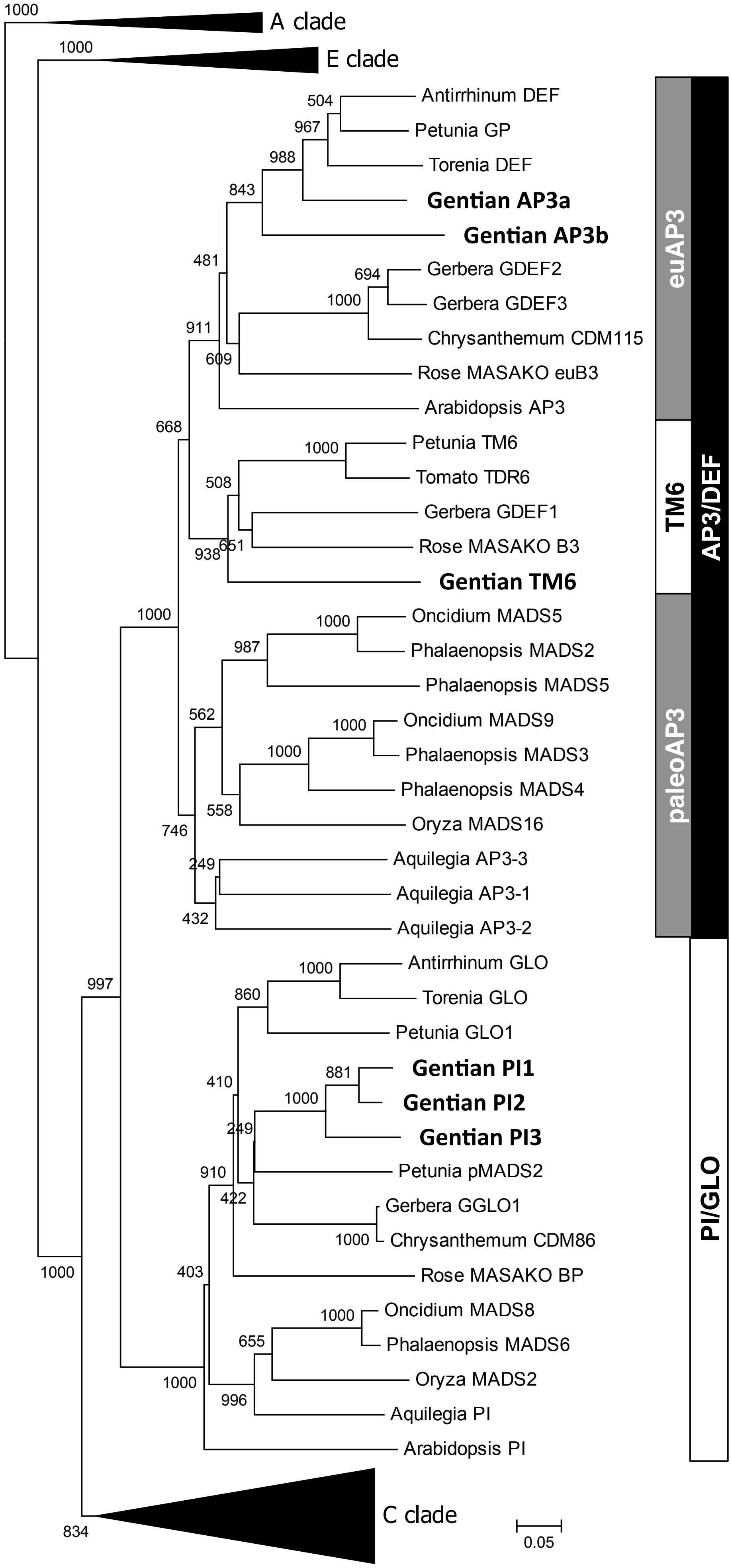

Supplementary Figure S3

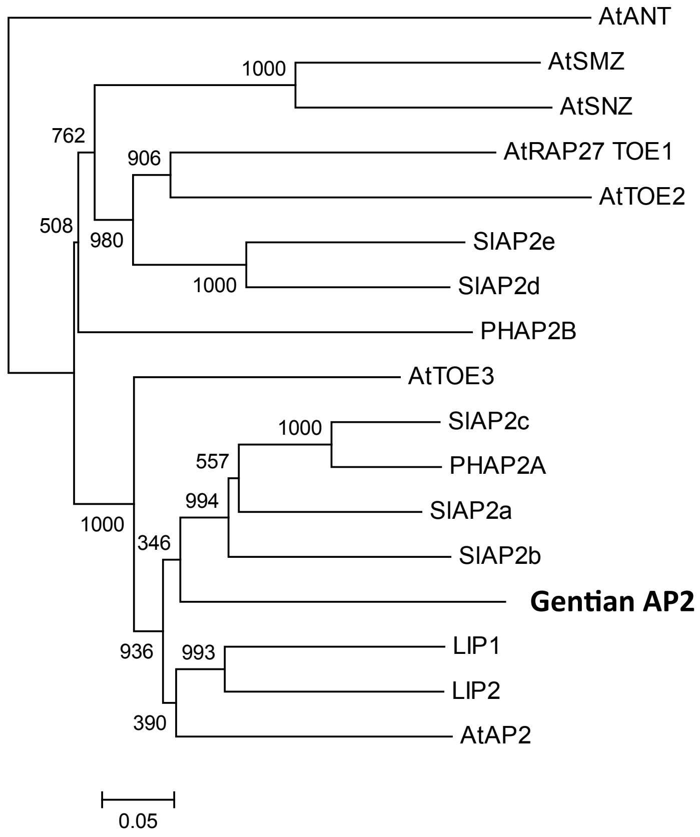

Supplementary Figure S4

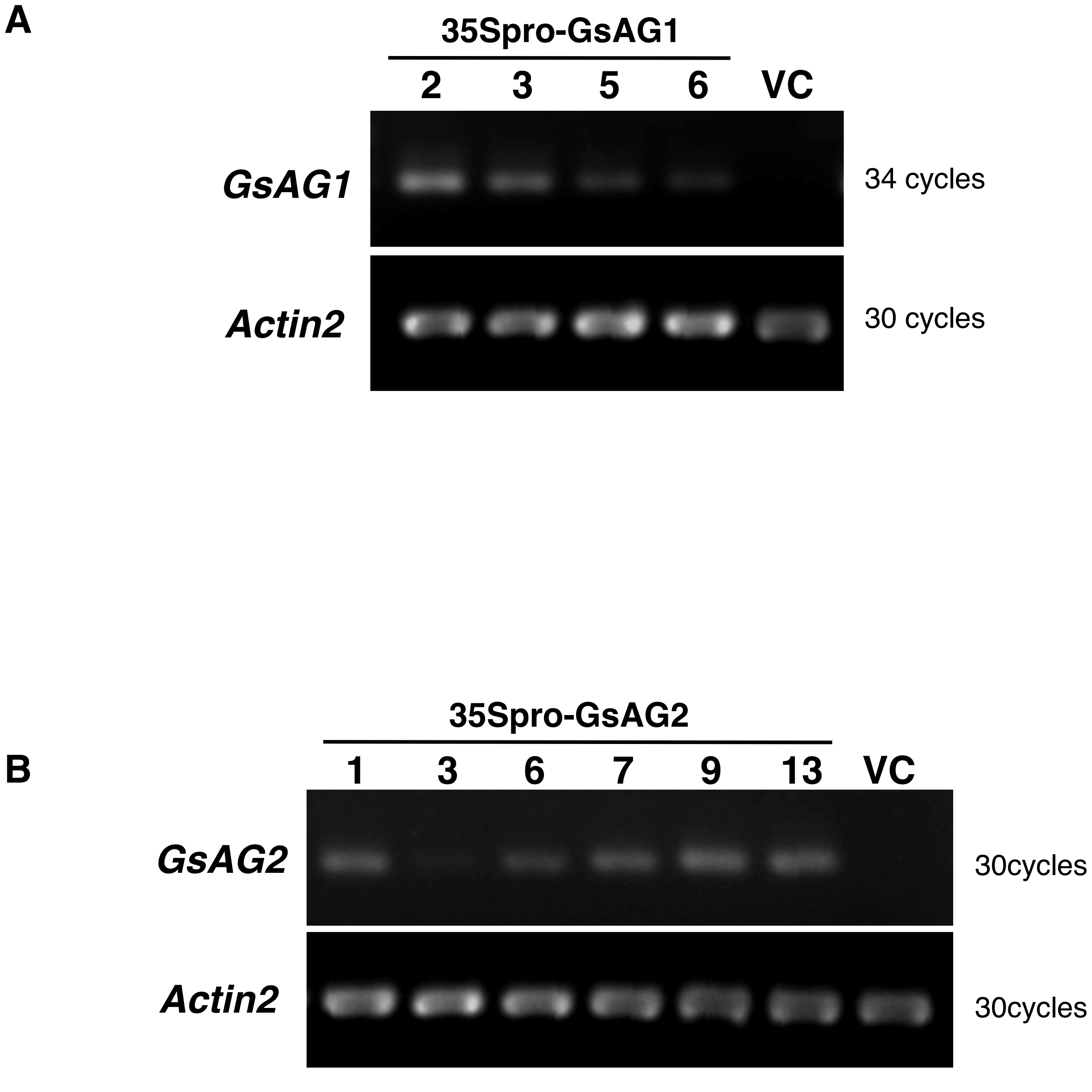

Supplement: Additional file 1: — Table S1. Sequences of primers used for RT-PCR and qRT-PCR analyses. Table S2. Sequences of primers used for ALSV vector construction. Figure S1. Phylogenetic tree of A- and E-class MADS-box proteins. Arabidopsis thaliana, AP1 (NP_177074), CAL (NP_564243), FUL (NP_568929), SEP1 (NP_568322), SEP2 (AAU82009), SEP3 (NP_564214), SEP4 (NP_178466); Antirrhinum majus SQUA (CAA45228); Gentiana scabra GsAP1 and GsFUL (this study); Petunia hybrida FBP2 (Q03489) and FBP5 (AAK21248). Phylogenetic tree was constructed using the neighbor-joining method with ClustalW and visualized using MEGA6. Numerals beside branches indicate bootstrap values from 1,000 replicates. Scale bar indicates 0.05 amino acid substitutions per site. Figure S2. Phylogenetic tree of B-class MADS-box proteins. Phylogenetic tree was constructed by the neighbor-joining method using ClustalW and visualized using MEGA6. Genbank accession numbers of amino acid sequences used in phylogenetic analysis are as follows: Antirrhinum majus DEF (P23706), GLO (Q03378); Arabidopsis thaliana, AP3 (AAD51903), PI (AAD51984); Chrysanthemum × morifolium CDM86 (AA022986), CDM115 (AA022985); Gentiana scabra AP3a, AP3b, PI1, PI2, PI3, TM6 (this study); Gerbera hybrida GDEF1 (Q9ZS28), GDEF2 (Q9ZS27), GDEF3 (ACV53813), GGLO1 (Q9ZS26); Onchidium hybrida MADS3 (AA045824), MADS5 (ADJ67234), MADS8 (ADJ67236), MADS9 (ADJ67235); Oryza sativa MADS2 (AAB52709), MADS16 (Q944S9); Petunia × hybrida GLO1 (AAS46018), GP (CAA49567), pMADS2 (QO7474); Phalaenopsis equestris MADS2 (AAR26628), MADS3 (AAR26629), MADS4 (AAR26626), MADS5 (AAR26627), MADS6 (AAV83997); Rosa rugosa MASAKO B3 (BAB63261), MASAKO BP (BAB11939), MASAKO euB3 (BAC79180); Solanum lycopersicum TDR6 (XP_004232453); Torenia fournieri DEF (BAG24492), GLO (BAG24493). Numerals beside branches indicate bootstrap values from 1,000 replicates. Scale bar indicates 0.05 amino acid substitutions per site. Figure S3. Phylogenic tree constructed using deduced amino acid sequences of GsAP2 and [file 12870_2015_569_MOESM1_ESM.pdf]
